# Performance Evaluation of STARPAM Polymer and Application in High Temperature and Salinity Reservoir

**DOI:** 10.1155/2018/9653953

**Published:** 2018-12-18

**Authors:** Chengli Zhang, Peng Wang, Guoliang Song

**Affiliations:** ^1^College of Petroleum Engineering, Northeast Petroleum University, Daqing, Heilongjiang 163318, China; ^2^College of Mathematics and Statistics, Northeast Petroleum University, Daqing, Heilongjiang 163318, China

## Abstract

Based on the properties of high temperature and salinity reservoir, the water-soluble polymer with good heat resistance and salt tolerance can be obtained through copolymerization between 2-acrylamide-2-methyl sulfonate monomer (AMPSN) and acrylamide monomer (AM) in water. The star shaped stable complexes (STARPAM) with the star nucleus of *β*-CD are prepared by living radical polymerization, which can improve the viscosity and change the percolation characteristics of the polymer in porous media. In the article, the performance of the STARPAM (star-shaped polymer) with heat resistance and salt tolerance was evaluated by comparing the viscosification property, heat and salt resistance, calcium and magnesium tolerance, and long-term thermal stability of STARPAM (star-shaped polymer) with those of HPAM (partially hydrolyzed polyacrylamide) and MO-4000 (linear polymer). The results of physical simulation experiment showed that the viscosity of the STARPAM is 3.3 times that of MO-4000 and 4 times that of HPAM under the conditions of mineralization degree of 20000 mg/L, concentration of 1500 mg/L, and 75°C, which indicated that heat resistance and salt tolerance of the STARPAM are excellent. Oil displacement experiments showed that STARPAM can enhance oil recovery by 20.53% after water flooding, and the effect of oil displacement is excellent. At present, 19 wells were effective with a ratio of 95.2%. Compared with before treatment, the daily liquid production increased by 136 m^3^, daily oil production increased by 44.6 t, water cut decreased by 4.67 percentage points, and flow pressure decreased by 1.15 MPa.

## 1. Introduction

The heterogeneity of the reservoir and the unfavorable mobility ratio are two important factors that affect the sweep efficiency and oil recovery of the water flooding. In the middle and later stage of water flooding, there is a problem of development that the injected water moves along the high permeability layers, while the utilization degree of the low permeability layers reduced, which could result in low recovery. HPAM is a linear water-soluble polymer, which is one of the most widely used water-soluble polymers. At present, it is widely used in oil field for tertiary recovery. The practice of field development has proved that polymer flooding is an effective method of improving oil recovery (EOR) and has become an important part of oil production in middle and later stage [1–3]. In actual development, it is found that, with the increase of temperature and salinity of the reservoir (Table 1), the electrical properties of the sodium carboxyl group in HPAM molecules are shielded, and the HPAM molecules are curly so that the ability of increasing viscosity declines [4–6]. When the content of Ca^2+^ and Mg^2+^ is higher, and the degree of hydrolysis of polyacrylamide is more than 40%, molecules of HPAM will combine with Ca^2+^, Mg^2+^, and other polyvalent ions, resulting in flocculation and sedimentation. The stability of the polymer is very important due to the long period of tertiary oil recovery. Therefore, hydrolysis degree of the polymer molecules used for tertiary recovery must be less than 40%. Only in this way can the polymer have the characteristics of heat resistance and salt tolerance in oil field application. However, the hydrolysis reaction of an amide group in HPAM is very rapid under acid and alkaline conditions, and the rate of hydrolysis under neutral conditions is accelerated with the increase of temperature, which makes HPAM do not have the characteristics of heat resistance and salt tolerance [7–9].

The polymers with heat resistance and salt tolerance for tertiary oil recovery have been developed at home and abroad, including HPAM of super-high molecular weight, amphoteric ion polymer, monomer copolymer, hydrophobically associating polymer, multiple composite polymer, comb polymer, and star-shaped polymer. By analyzing the mechanism of heat resistance and salt tolerance, it is considered that star-shaped polymers are the most promising, if the star-shaped polymers with function of heat resistance and salt tolerance are developed, which can provide theoretical basis and technical support for tertiary recovery to enhance oil recovery [10, 11].

Flory P J [12] put forward the concept of star-shaped polymer in 1948; unlike linear straight chain polymers, it is a type of polymer that several or more polymer chains can be produced from one fulcrum or nucleus. In 1950s, Flory P J [13] proposed the idea of synthesizing highly branched polymers with star structures with ABn monomers and predicted the parameter of relative molecular mass distribution of polymers and so on. In 1956, Morton M et al. [14, 15] synthesized four-arms star-shaped polymers with tetrachlorosilane as the core and straight chain polystyrene as the arm. Because of their branching structure and dispersion, this kind of chemical agent has special properties and functions, which has become a research focus of star-shaped polymers. In 1985, Tomalia DA and Newkome XS et al. [16] published research achievements on the dendritic supramolecules synthesized by diffusion outward from a core, which opened a new field for the research of star-shaped polymers. At present, the commonly used polymerization methods for preparing star-shaped polymers include atom transfer radical polymerization, reversible addition fragmentation chain transfer polymerization, and ring opening polymerization [17, 18]. Wei Ding [19] and Ying Sun et al. [20] synthesized star-shaped polyacrylamide through the methods of “COREFIRST” and single electron transfer radical polymerization. Fuxiao Wang [21] synthesized a series of star-shaped hydrophobically associating polyacrylamide with different content of ODAC by photoinitiated radical polymerization.

*β*-CD is a kind of natural macromolecules linked by glucan; large amounts of hydroxyl at both ends can be directly modified and used as macromolecular initiators [22]. The star-shaped polymer with core of *β*-CD has more abundant properties and applications than ordinary cross-linked star-shaped polymers. The molecular structure of *β*-CD includes polyhydroxy and hydrophobic cavities, and the properties of polyhydroxyl groups determine that *β*-CD can be used as nuclei of the star-shaped polymers. What is more important is that the hydrophobic cavities of *β*-CD have supramolecular inclusion for a wide range of guest molecules, which can change the seepage characteristics of the star-shaped polymers in reservoirs [22–24]. Through the active free radical polymerization, a stable star-shaped complex with *β*-CD as the core is formed, which can improve the viscosity and change the seepage characteristics of the polymer in porous media. The existence of multifunctional monomers in STARPAM polymer ensures that the hydrolysis of star-shaped polymer can be limited under the conditions of high temperature and high salinity, and the star-shaped structure can increase the structural regularity of polymer molecular cluster and be with temperature and salt tolerance characteristics.

## 2. Mechanism of Synthesis of a New Type of Heat Resistance and Salt Tolerance Polymer (STARPAM)

### 2.1. The Method of Synthesis of STARPAM

**Figure 1:**
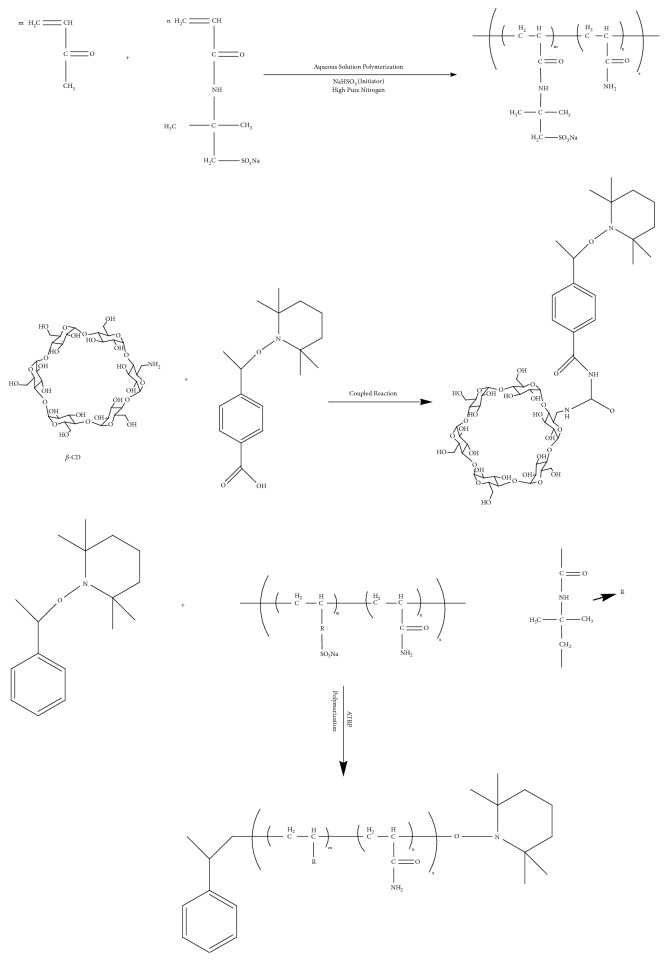
Synthesis of STARPAM with a nucleation of *β*-CD.

The water-soluble polymer with good characteristics of heat resistance and salt tolerance can be obtained by aqueous copolymerization of 2-acrylamide-2-methyl sulfonate monomer (AMPSN) and acrylamide monomer (AM). *β*-CD is modified as a seven-membered functional initiator by 2,2,6,6-tetramethylpiperidinyloxy-TEMPO [25]. The stable star-shaped complex (STARPAM) with the nucleus of *β*-CD (*β*-cyclodextrin) is formed through TEMPO-mediated reactive radical polymerization; on this basis, the viscosity of polymer is increased and the percolation characteristics of the polymer in porous media are changed, as shown in Figure 1.

### 2.2. Steps of the Synthesis of STARPAM

(1) The quantitative 2-acrylamide-2-methylsulfonate monomer (AMPSN) was added to wide-mouth bottle, which was dissolved with a moderate amount of deionized water, neutralizing with sodium hydroxide in the ice water bath until to pH of 7~8, and slowly stirring to completely dissolve.

(2) The PAM was precipitated with anhydrous alcohol for several times and then dried in vacuum. A certain amount of PAM was accurately weighed and dissolved in appropriate amount of deionized water, when the solution reached the expected value and was placed in a wide-mouth bottle.

(3) Input the nitrogen after the above solution was sealed, and the weighed sodium bisulfite and potassium persulfate solution are added in this order using a pipette (respectively, prepare the deionized water solution of a certain concentration in advance).

(4) Sealing and the nitrogen were continued input, and then the wide-mouth bottle was put in the water bath with constant temperature, and the reaction time is specified. After the end of the reaction, the product of colloidal sulfonated polyacrylamide was obtained.

(5) 2,2,6,6-Tetramethylpiperidinyloxy benzoic acid (3.4 g, 9.4 mmol) was coupled with *β*-CD (*β*-cyclodextrin 8.6 g, 7.0 mmol) using dicyclohexylcarbodiimide (DCC 2.1 g, 9.5mmol) in DMF (dimethylformamide 100 mL). To couple the carboxylic acid exhaustively with the amino groups, the reaction was performed in the presence of N-hydroxybenzotriazole (HBT 2.0 g, 12.1 mmol) and triethylamine (Et3N 1.01 g, 9.0 mmol).

(6) Initiator (2.2 g, 0.70 *μ*mol) was dissolved in sulfonate polyacrylamide (15 g, 120 mmol). Oxygen was removed from the solution by freezing in liquid nitrogen, evacuating the flask, warming to room temperature, and flushing the flask with argon gas. This procedure was repeated three times. The mixture was then stirred at 120°C for 6 h. After cooling in liquid nitrogen, the mixture was diluted with chloroform (25 mL) and then poured into methanol (1.5 L). The precipitate was filtered off and purified by reprecipitation with chloroform-methanol and dried in vacuo to give polymer as a white powder.

## 3. Heat Resistance and Salt Tolerance Performance Evaluation of STARPAM

### 3.1. Experimental Equipment and Reagents

(1) SNB-2 Digital Viscometer (Shanghai Jingke day U.S. Trade Co. Ltd.); speed is 6 r/min.

(2) HPAM, relative molecular mass 12 million, Karamay Xinke chemical (Group) Co., Ltd.; the degree of hydrolysis is 22.3%.

(3) MO-4000, relative molecular mass > 20 million, Karamay Xinke chemical (Group) Co., Ltd.; the degree of hydrolysis is 22.8%.

(4) STARPAM, relative molecular mass > 25 million, the degree of hydrolysis is 32.5%, and the content of *β*-CD (mass fraction) is 0.065%.

### 3.2. The Performance of Viscosity Increasing and Viscosity-Temperature

**Figure 2:**
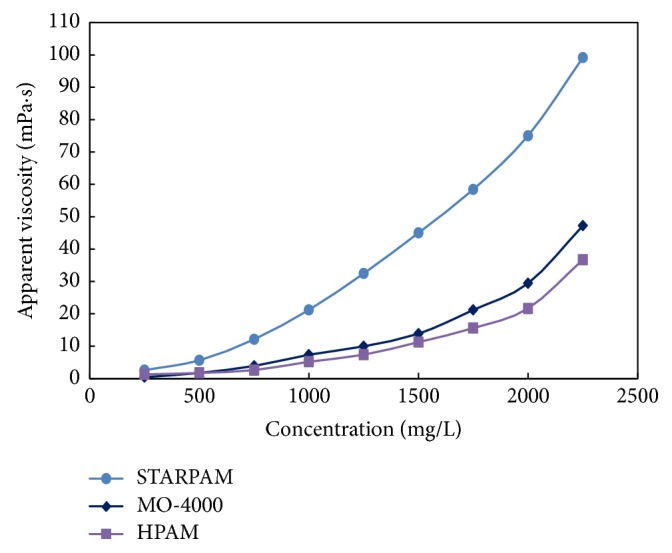
Comparison of adding viscosity.

At 75°C, the concentration of polymers was changed with 250 mg/L as the concentration step, and the effect of solution concentration on the viscosity of polymers was observed. The results were shown in Table 2 and Figure 2.

It can be seen from Table 2 and Figure 2 that the viscosity of polymer solution increases with the increase of the concentration. When the concentration reaches 1500 mg/L, the viscosity of STARPAM solution is 3.3 times of HPAM polymer solution, and the performance of viscosity increasing is obviously superior to the other two linear polymers. When reaching the same viscosity, the content of STARPAM polymer is lower, which can reduce the cost of oil production. The STARPAM contains more than 7 long chain arms, with large viscosity and molecular weight of that is more than 25 million, which can increase the volume of the hydrodynamics of the molecular chain. The viscosity of the polymer solution is relatively higher under the same polymer concentration.

**Figure 3:**
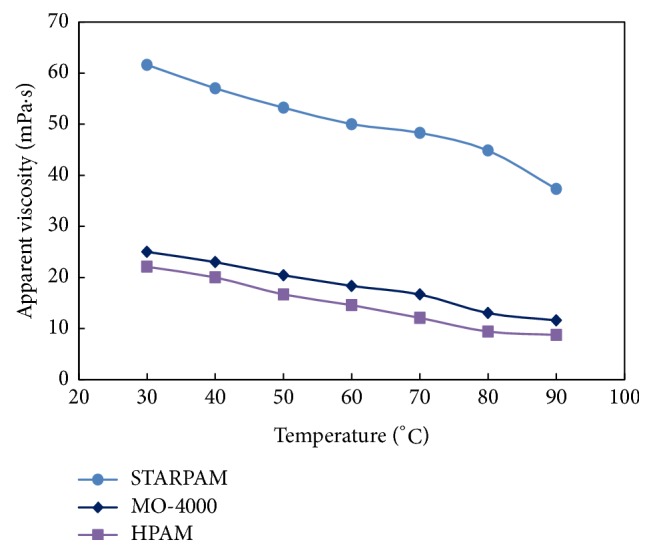
Comparison of heat resistance.

When the concentration of polymer solution remained of 1500 mg/L and the salinity remained of 20000 mg/L, changing the temperature on the basis of the temperature step with 10°C, the effect of temperature on the viscosity of polymer solution was observed. The results were shown in Table 3 and Figure 3.

It can be seen from Table 3 and Figure 3 that the viscosity of polymer solution gradually decreases with the increase of temperature, and the heat resistance of STARPAM polymer solution is the best, which indicated that the displacement viscosity of the leading edge of the series of polymer is high. The main chains of polymer molecules changing into stars can effectively increase the rigidity of molecular chain and the regularity of molecular structure, which makes the polymer molecular chain crimp difficult and the hydraulic radius of molecular chain rotation increase. Therefore, viscosity of STARPAM is high under high temperature.

The adding of sulfonic acid groups on polyacrylamide molecules can enhance the polarity of molecules and form hydrogen bonds between macromolecules, and the long chain of carbons in the group also can enhance the rigidity of the molecule; thus the heat resistance is increased.

### 3.3. The Performance of Ca^*2+*^, Mg^*2+*^, and Salt Tolerance

**Figure 4:**
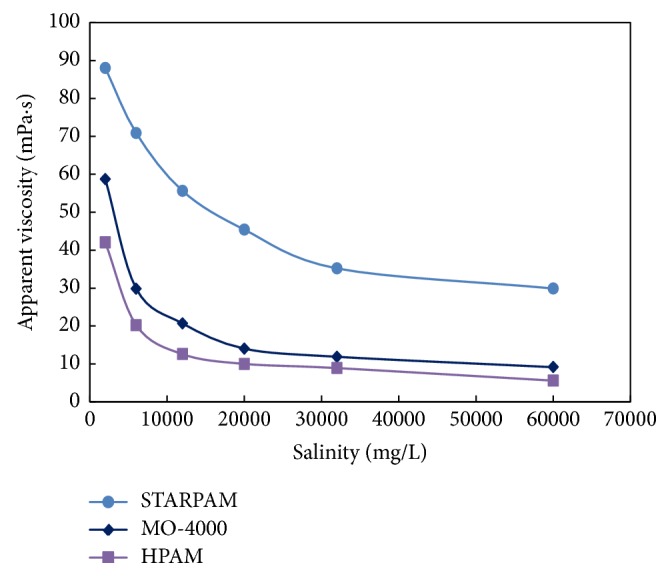
Comparison of the influence of polymers viscosity on the salinity.

Three kinds of polymer solutions with different mineralization degree were prepared respectively, and the effects of different salinity on the viscosity of polymer solution were observed to verify the salt resistance. The results were shown in Table 4 and Figure 4.

It can be seen from Table 4 and Figure 4 that the viscosity of polymer solution decreases with the increase of salinity. When the salinity is less than 12000 mg/L, the viscosity of STARPAM solution is slower than other two solutions, and the salt sensitivity is relatively lower. When the salinity is more than 12000 mg/L, the viscosity of STARPAM solution is higher, which indicated that the viscosity of the three polymer solutions decreases slowly, and the performance of salt resistance of STARPAM polymer is well.

**Figure 5:**
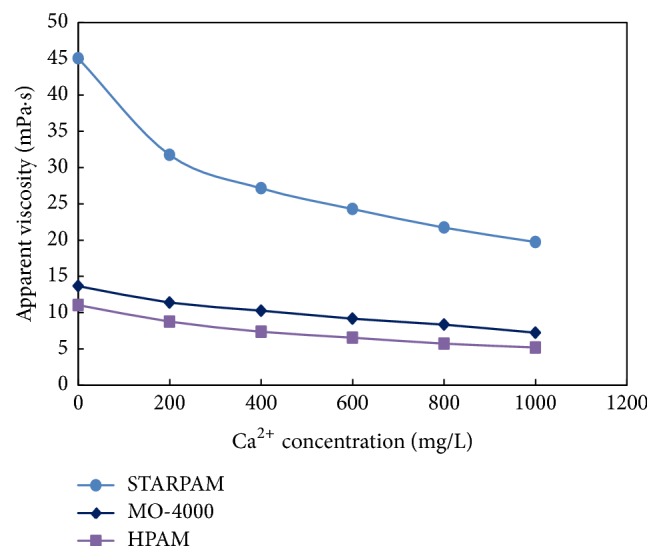
Comparison of the influence of polymers viscosity on the Ca^2+^.

The polymer solutions with different concentration of Ca^2+^ were prepared. The effect of Ca^2+^ concentration on the viscosity of polymer solutions was observed. The results were shown in Table 5 and Figure 5.

As seen from Table 5 and Figure 5, with the increase of Ca^2+^ concentration in polymer solutions, the viscosity of polymer solutions gradually decreases, and the performance of Ca^2+^ resistance of STARPAM polymer solution is the best. After introducing the strong anion groups in STARPAM chain, the structure contains strong anionic, water-soluble sulfonic groups, screened amido groups, and unsaturated double bond, so that it has excellent performance. The sulfonic groups can effectively inhibit the hydrolysis of the amide groups and has a good tolerance to the two valence cations, which will not react with the two valence ions to produce precipitation and enhance the salt resistance.

### 3.4. The Performance of Stability

**Figure 6:**
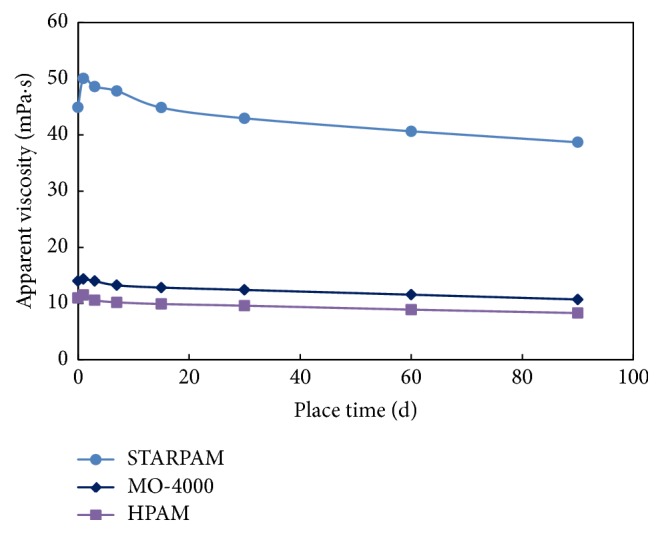
The relationship between three kinds of polymer viscosity and aging time (75°C, high purity nitrogen and vacuum deoxidization).

**Figure 7:**
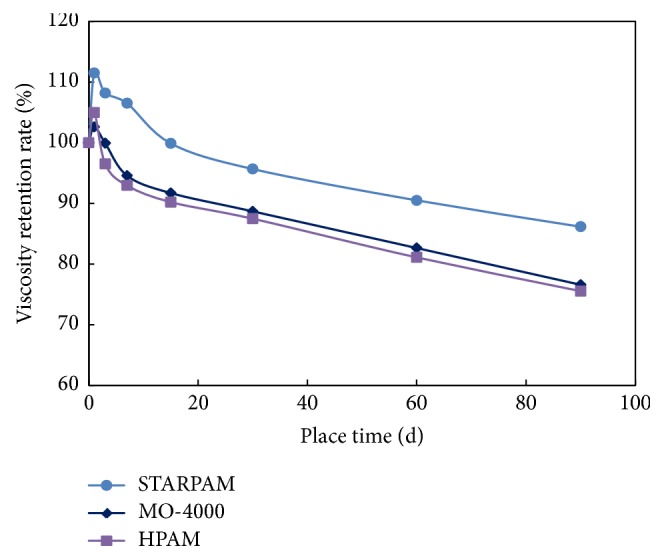
The relationship between viscosity retention rate and aging time of three polymers (75°C, high purity nitrogen and vacuum deoxidization).

The relationship between the apparent viscosity of the three polymers and the aging time was recorded under the conditions of high purity nitrogen and vacuum deoxidization at 75°C, seen as Table 6 and Figure 6, and the retention rate of viscosity was calculated as shown in Table 7 and Figure 7.

The viscosity of the three kinds of polymer solution decreases with the increase of aging time. It is because that polymer chain breaks down and degrades due to high temperature aging, and the interaction between polymer molecules is weakened and the viscosity is reduced. The shielding effect of salt makes the STARPAM polymer chain curl, which effectively protects the main chain when aging at high temperature, thus slowing down the degradation.

The viscosity retention rate of the three polymers is over 90% in the first 30 days. The viscosity retention rate of STARPAM polymer solution is 7.4% higher than HPAM polymer and is 9.2% higher than MO-4000 polymer, indicating that the stability of STARPAM polymer solution is better.

## 4. Percolation Characteristics Evaluation of STARPAM

The core that gas measurement permeability 1450~1500 × 10^-3 ^*μ*m^2^, length 5.00~8.00 cm, and diameter 2.50 cm was selected. According to “standard Q/ SDY 1119-2003 implementation rule 6.19”, under the conditions of the salinity of compound salt water 19334 mg/L, the concentration of polymer, respectively, 800 mg/L, 1000 mg/L, and 1500 mg/L, temperature of 75°C, and injection rate of 0.50 mL/min, the resistance coefficient and residual resistance coefficient of the new polymer samples were measured.

**Figure 8:**
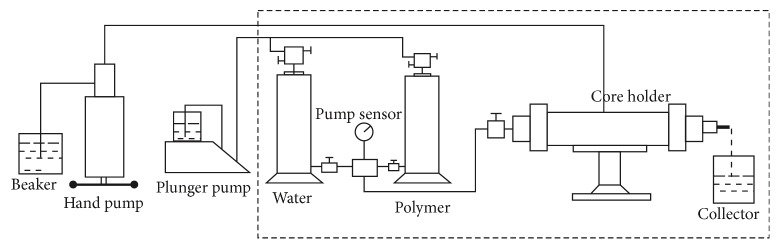
The flow characteristics evaluation process.

**Figure 9:**
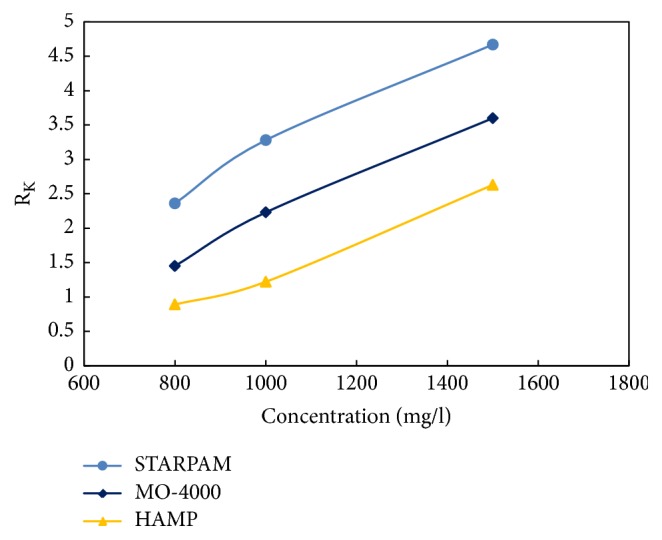
Resistance coefficient of three polymers.

**Figure 10:**
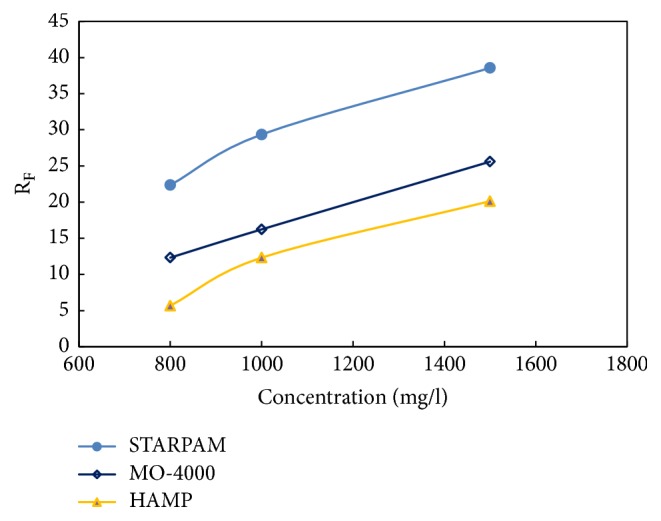
Residual resistance coefficient of three polymers.

Experimental steps are as follows: (1) injecting 19334 mg/L compound salt water until the pressure is stable; (2) injecting new polymer solution to flood oil until the pressure is stable (the new polymer solution was filtered by a sand core funnel of G2 type before injection); (3) injecting 19334 mg/L compound salt water until stable pressure. The experimental process is shown in Figure 8 and data are presented in Table 8 and Figures 9 and 10.

From Table 8 and Figures 9-10, it is shown that the resistance coefficient and the residual resistance coefficient increase with the increase of the concentration of the polymer solution for the same molecular weight, and whatever the core and the concentration, which of STARPAM polymers are higher than those of other two kinds of polymers.

The resistance coefficient and residual resistance coefficient are generated through the capture and retention of polymer solution flowing in porous media. The trapped polymer molecules are strongly resistance to water and relatively weakly resistance to oil. Therefore, the original percolation characteristics and flow channels of the reservoir will be greatly changed, and the permeability will be reduced.

## 5. Oil Displacement Performance Evaluation of STARPAM

**Figure 11:**
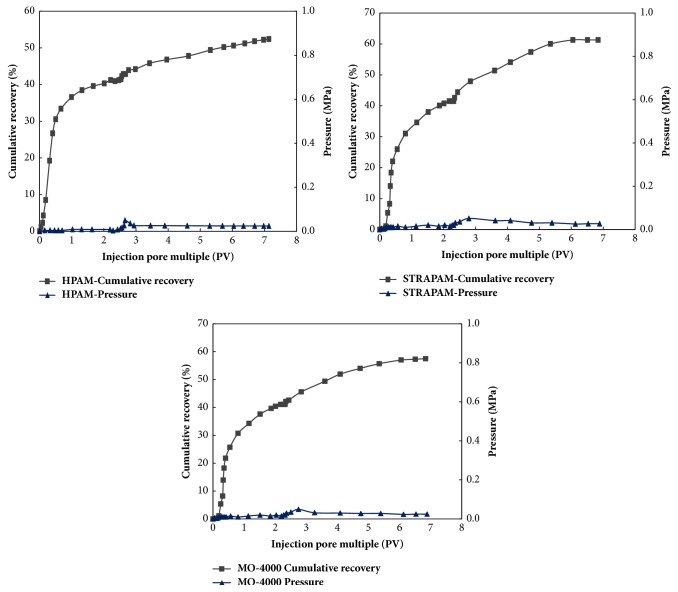
Polymer flooding curve.

(1) Cemented core: permeability of core is 1450~1500x10^−3^um^2^.

(2) Simulated oil: the mixture of oil and kerosene in SL oil field with the ratio of 5:1, the viscosity is 24.2 mPa·s at 75°C, and the viscosity of crude oil is 97.7 mPa·s at 75°C.

(3) Experimental steps: ① measuring permeability of core with water and saturating simulated oil; ② water flooding to water cut of 98%;  ③ polymer flooding (0.2 PV); ④ following up water flooding (water cut 98%). The experimental data are presented in Table 9 and Figure 11.

The effect of three kinds of polymer flooding is shown in Table 9. The oil displacement efficiency of STARPAM polymer is relatively higher, and the recovery rate is 20.53%, the other two kinds of polymer flooding efficiency are lower, and the recovery rate is 17.13% and 12.09%, respectively. From Figure 11, we can see that the oil recovery at the end stage of water flooding is almost constant, but increasing after polymer flooding, of which the STARPAM polymer is obviously the best. The efficiency of polymer flooding depends on the difference of viscosity. According to the analysis of the performance of the three kinds of polymer, the viscosity of STARPAM polymer is the highest, so that the effect of oil displacement is the best.

It can be seen from the experimental results that, for every kind of polymers, the injection pressure increases with the increase of polymer injection volume and then be stability. Although the molecular weight of star-shaped polymers is much higher than that of the others, the injection pressure is similar to that of MO-4000 linear polymer, and the injection ability is good. It is because the conical structure of star nuclear *β*-CD is hydrophilic to the outer cavity and hydrophobic to the inner cavity. The hydrogen bond formed between the hydroxyl group on the *β*-CD and the water molecules makes the *β*-CD water-soluble. Unique cavity structure of *β*-CD can make matching between subject and object. Through action of noncovalent bonding, a stable complex with hydrophobicity, certain shape, and suitable size can be formed to affect the behavior of polymer solution and change its seepage characteristics in porous media.

## 6. Field Application

### 6.1. Block Overview

The area of SL-A block in SL high temperature and salinity oilfield is 0.48 km^2^, and the underground pore volume is 102.3 × 10^4^ m^3^. The geological reserve of target stratum is 65.2 × 10^4^ t, the average shooting sandstone thickness in single well is 12.32 m, the effective thickness is 10.14 m, and the average effective permeability is 230.6 × 10^-3 ^*μ*m^2^. Five-point well pattern is adopted in A block, and there are 33 wells, including 13 injection wells, 20 production wells; the distance between injection and production wells is 120 m. The average temperature of the reservoir is 75°C, and the total salinity of formation water is 15167 mg/L. SL-A block is a typically reservoir with high temperature and salinity, but it has potential for development.

### 6.2. Analysis of Field Application Effect

In order to reduce formation temperature and ion interference, blank water flooding was conduct of SL-A block in January 2015. Star-shaped polymer injection system was adopted in the initial stage of polymer flooding in August 2016, and the speed of the high and low concentration of polymer injection was kept at 0.18 ~ 0.19 PV/a; the injection speed remains around 0.20 ~ 0.22PV/a in the concentration reduction and acceleration stage, the average injection concentration was 1596 mg/L, and the total amount of polymer was 1050 mg/ L·PV.

**Figure 12:**
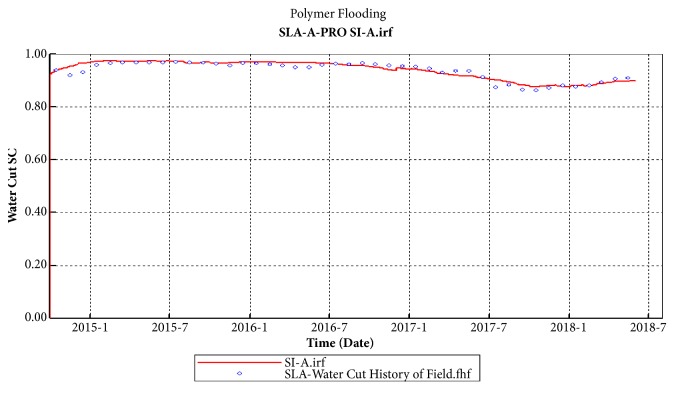
SL-A block comprehensive water cut curve.

**Figure 13:**
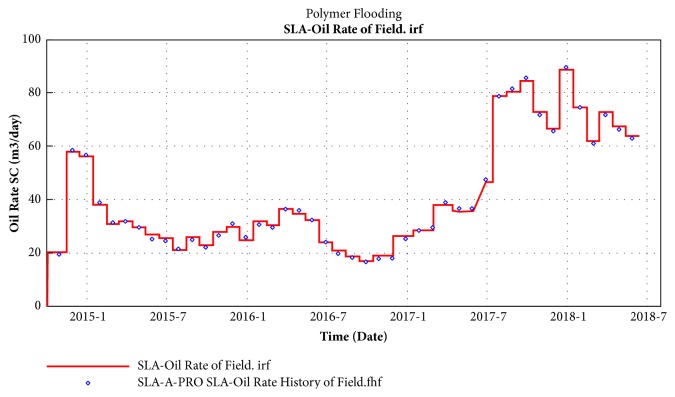
SL-A block daily oil production curve.

**Figure 14:**
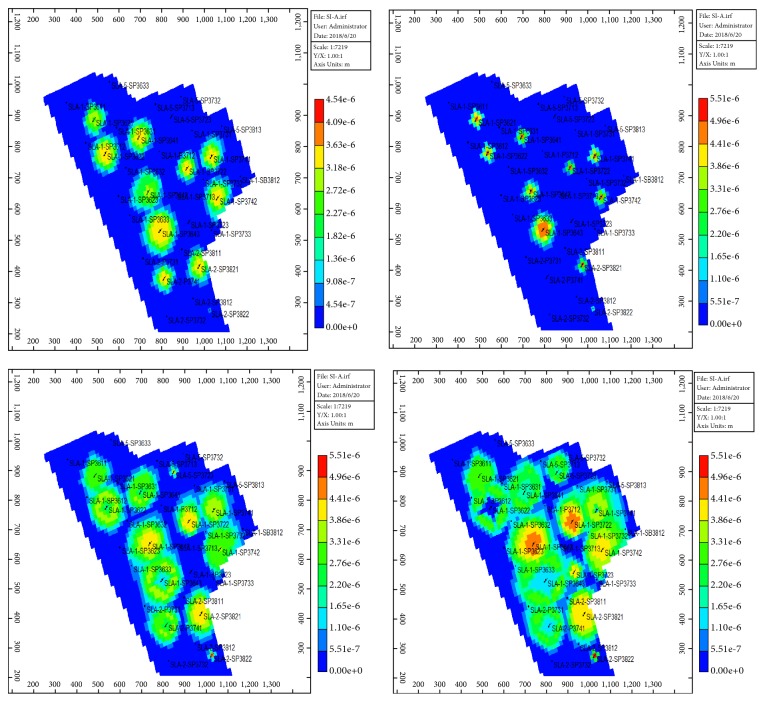
Distribution map of polymer concentration field in SL-Ι layer.

**Figure 15:**
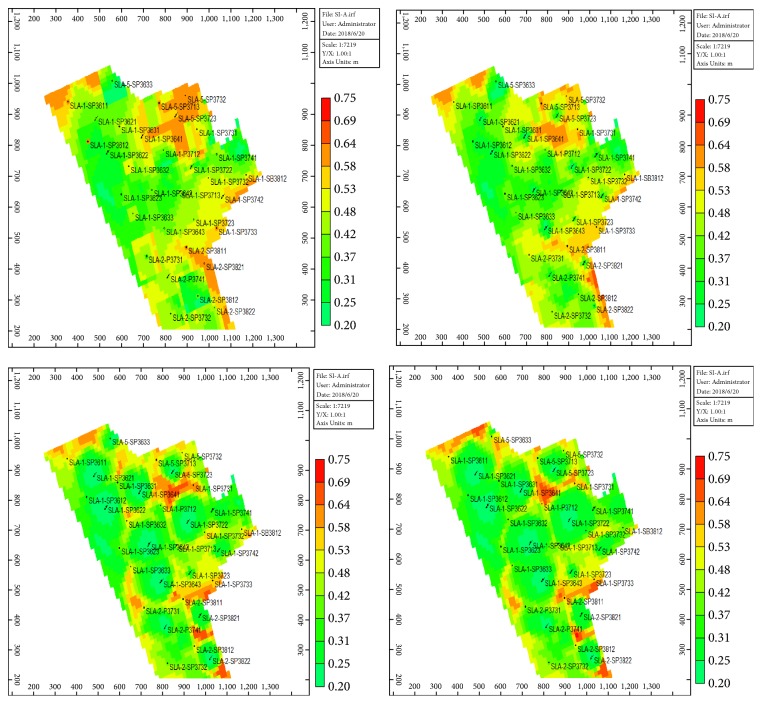
Distribution map of residual oil in SL-Ι layer.

Block SL-A entered the low-water-cut period in August 2017 and water cut reached the lowest value of 87.8% in November 2017. At present, 19 wells have been effective with a ratio of 95.2%. Compared with before, daily liquid production increased by 136 m^3^, daily oil production increased by 44.6 t, water cut decreased by 4.67 percentage points, and flow pressure decreased by 1.15 MPa. The water cut and daily oil production for SL-Ι layers of SL-A block are shown as Figures 12 and 13. The polymer concentration and residual oil are presented in Figures 14 and 15. These two indicators are tested every six months, and the time points are October 2016, April 2017, October 2017, and April 2018, respectively.

As the polymer-slug gradually approaching the oil wells, the polymer concentration in the polymer injection wells in the SL-Ι layers became lower, and the polymer accumulated between the oil wells and the polymer injection wells, and the remaining oil was driven to the bottom of the oil wells along the main line. It is proved that the percolation performance of the STARPAM polymer is good and the sweeping range is wide. It can be used in the high temperature and salinity reservoirs to enhance oil recovery.

## 7. Conclusions

(1) Under the condition of high temperature and salinity, the rupture of the molecular chain will reduce the polymer degradation viscosity; the main chains of polymer molecules changing into stars can effectively increase the rigidity of molecular chain and the regularity of molecular structure, which makes the of polymer molecular chain crimp difficult and the hydraulic radius of molecular chain rotation increase. Therefore, viscosity of STARPAM is high under high temperature.

(2) The stable star-shaped complex (STARPAM) with *β*-CD (*β*-cyclodextrin) as the star nucleus is formed by polymerization of active radical, which can affect the behavior of polymer solution and change its seepage characteristics in porous media.

(3) Through a series of experiments, it is proved that the viscosity of STARPAM polymer solution is relatively higher under the same polymer concentration, the oil displacement efficiency is the best and the oil recovery is higher after water flooding.

(4) STARPAM polymer solution was injected to block SL-A firstly in August 2016, the period of low water cut was appeared in August 2017, and the smallest value of water cut reached 87.8% in November 2017. At present, 19 wells have been effective with a ratio of 95.2%. Compared with before, daily liquid production increased by 136 m^3^, daily oil production increased by 44.6 t, water cut decreased by 4.67 percentage points, and flow pressure decreased by 1.15 MPa.

## Figures and Tables

**Table 1:** Standard of oil layer division in an oil field.

Category	Screening criteria		
	Original formation temperature / °C	Formation water salinity / mg/L	Ca^2+^ and Mg^2+^ content / mg/L
Class I	≤70	≤10000	≤200
Class II	70-80	10000-30000	200-400
Class III	80-93	30000-1000000	≥400
Class IV	Oil layer is sanding seriously and has poor connectivity of the oil layer		

**Table 2:** Comparison of adding viscosity.

Concentration /(mg/L)	Apparent viscosity/(mPa·s)		
	STARPAM	MO-4000	HPAM
250	2.60	0.43	1.30
500	5.63	1.73	1.73
750	12.12	3.89	2.60
1000	21.20	7.36	5.19
1250	32.45	9.95	7.36
1500	45.00	13.85	11.25
1750	58.41	21.20	15.58
2000	75.00	29.40	21.63

**Table 3:** Comparison of heat resistance.

Temperature /(°C)	Apparent viscosity/(mPa·s)		
	STARPAM	MO-4000	HPAM
30	61.62	25.00	22.08
40	57.01	23.00	20.00
50	53.23	20.42	16.67
60	50.00	18.33	14.58
70	48.30	16.67	12.08
80	44.85	13.01	9.40
90	37.31	11.60	8.75

**Table 4:** Comparison of the influence of polymers viscosity on the salinity.

Salinity /(mg/ L)	Apparent viscosity /(mPa·s)		
	STARPAM	MO-4000	HPAM
2000	88.00	58.72	42.01
6000	70.84	29.83	20.20
12000	55.60	20.66	12.56
20000	45.41	14.02	10.00
32000	35.17	11.88	8.90
60000	29.89	9.13	5.59

**Table 5:** Comparison of the influence of polymers viscosity on the Ca^2+^.

Ca^2+^concentration /(mg/L)	Apparent viscosity /(mPa·s)		
	STARPAM	MO-4000	HPAM
0	45.06	13.66	11.05
200	31.74	11.39	8.77
400	27.14	10.28	7.37
600	24.29	9.17	6.55
800	21.72	8.35	5.74
1000	19.74	7.24	5.21
1200	45.06	13.66	11.05

**Table 6:** The relationship between three kinds of polymer viscosity and aging time.

Place time /(d)	Apparent viscosity /(mPa·s)		
	STARPAM	MO-4000	HPAM
0	44.91	13.99	10.98
1	50.06	14.35	11.52
3	48.58	13.98	10.59
7	47.83	13.23	10.20
15	44.85	12.83	9.90
30	42.96	12.40	9.60
60	40.64	11.56	8.90

**Table 7:** The relationship between viscosity retention rate and aging time of three polymers.

Place time /(d)	Viscosity retention rate /(%)		
	STARPAM	MO-4000	HPAM
0	100.00	100.00	100.00
1	111.47	102.62	104.96
3	108.18	99.92	96.49
7	106.50	94.56	92.93
15	99.88	91.69	90.20
30	95.65	88.68	87.47
60	90.49	82.63	81.09

**Table 8:** Resistance coefficient and residual resistance coefficient of three polymers.

Sample	Core parameters							R_F_	R_K_	C / mg/L
	Number	L / cm	R / cm	V / cm^3^	*ϕ* / %	K / 10^−3^*μ*m^2^	*μ* / mPa·s			
STARPAM	S-1	5.92	2.50	8.25	28.39	1476	18.6	22.35	2.36	800
STARPAM	S-2	5.99	2.51	8.33	28.16	1506	22.6	29.36	3.28	1000
STARPAM	S-3	5.92	2.50	8.19	27.89	1466	45.2	38.56	4.67	1500
MO-4000	S-1	5.92	2.50	8.25	28.39	1476	9.6	12.32	1.45	800
MO-4000	S-2	5.99	2.51	8.33	28.16	1506	10.3	16.23	2.23	1000
MO-4000	S-3	5.92	2.50	8.19	27.89	1466	14.8	25.60	3.60	1500
HAMP	S-1	5.92	2.50	8.25	28.39	1476	8.2	5.66	0.89	800
HAMP	S-2	5.99	2.51	8.33	28.16	1506	9.6	12.32	1.22	1000
HAMP	S-3	5.92	2.50	8.19	27.89	1466	12.6	20.13	2.63	1500

Remarks: L: length, R: diameter, V: pore volume, *ϕ*: porosity, K: permeability, *μ*: viscosity, R_F_: resistance coefficient, R_K_: residual resistance coefficient, and C: concentration.

**Table 9:** Comparison of oil displacement effect.

K / 10^−3^*μ*m^2^	Sample	*μ* / mPa·s	S_O_ / %	R_W_ / %	Polymer flooding	
					1500 mg/L, 0.2PV	
					R_P_ / %	R_E_ / %
1050	STARPAM	45.1	67.64	40.77	61.30	20.53
1039	MO-4000	14.5	66.67	40.37	57.50	17.13
1145	HPAM	11.3	66.79	40.31	52.40	12.09

Remarks: K: permeability, *μ*: viscosity, S_O_: oil saturation, R_W_: recovery of water flooding, R_P_: cumulative recovery, and R_E_: enhanced oil recovery of polymer flooding.

## Data Availability

The data used to support the findings of this study are available from the corresponding author upon request.
